# Some Computational Aspects of the Brain Computer Interfaces Based on Inner Music

**DOI:** 10.1155/2009/950403

**Published:** 2009-05-26

**Authors:** Wlodzimierz Klonowski, Wlodzisław Duch, Aleksandar Perovic, Aleksandar Jovanovic

**Affiliations:** ^1^Laboratory of Biosignal Analysis Fundamentals, Institute of Biocybernetics & Biomedical Engineering, Polish Academy of Sciences, 02109 Warsaw, Poland; ^2^Department of Informatics, Nicolaus Copernicus University, 87-100 Torun, Poland; ^3^Group for Intelligent Systems, School of Mathematics, University of Belgrade, 11000 Belgrade, Serbia

## Abstract

We discuss the BCI based on inner tones and inner music. We had some success in the detection of inner tones, the imagined tones which are not sung aloud. Rather easily imagined and controlled, they offer a set of states usable for BCI, with high information capacity and high transfer rates. Imagination of sounds or musical tunes could provide a multicommand language for BCI, as if using the natural language. Moreover, this approach could be used to test musical abilities. Such BCI interface could be superior when there is a need for a broader command language. Some computational estimates and unresolved difficulties are presented.

## 1. Introduction

The recent impressive developments of brain computer interfaces, BCI, after initial great success, especially, by the group of Babiloni [1–5], and earlier biofeedback achievements [6], open room for optimism in diverse directions. Work on BCI has been concentrated on motor imagery; here an alternative direction is proposed, musical imagery [7, 8].

Just like an imagination of hand or finger movement is related to changes in activity of the brain somehow resembling those connected with the real movement, so the process of mental hearing and comprehending music is related to changes in brain activity somehow resembling those occurring in the brain when listening to real physical sounds of music. Such a cognitive process of *auditory imagery*, of singing in the mind, is also called *audiation*; audiation of music is analogous to thinking in a language. We propose that it is possible to construct a BCI based on the Inner Tones and Inner Music, that is, the BCI in which discrimination of the imagined or inner tones is used as the basic brain signal for the formation of the BCI set of commands—musical language. 

**Figure 1:**
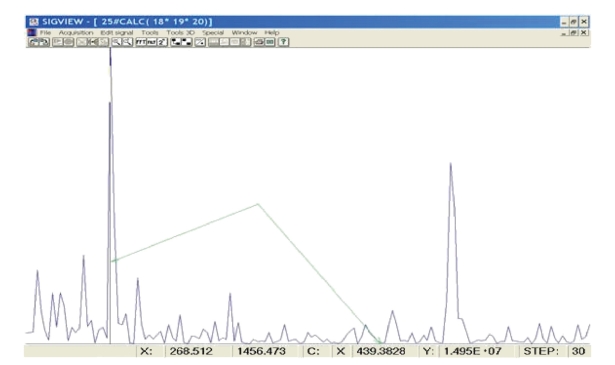
A part of a power spectrum of EEG recording of sequentially played tones d and a, marked the spectral line corresponding to the tone a.

**Figure 2:**
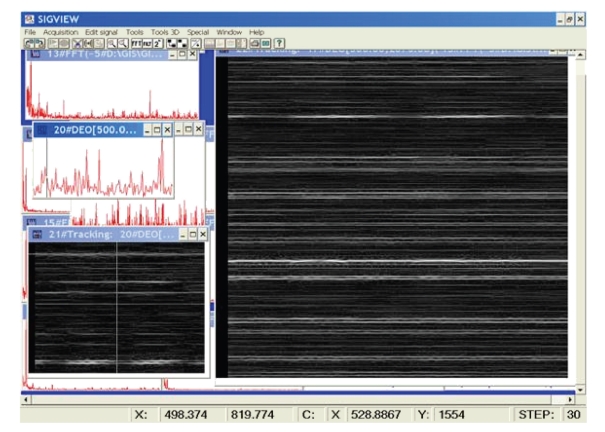
A power spectrum of EEG signal recording with simultaneously played tones c2 and g2, top left; its part containing c2 and g2 lines, left center; the spectrogram of the extracted portion of the spectrum, with prominent c2 and g2 lines, low left; the major part of the spectrogram exhibiting some artifacts and other high-frequency features, right side. Low frequency—bottom; intensity—brightness coded; time—recent at the right edge.

After partial success in the identification of inner tones, as reported in [7, 9–13], in spite of encountering serious difficulties, we propose that more attention should be given to the BCI based on the Inner Tones and Inner Music. We have developed systems for the real-time acquisition and analysis of unlimited number of EEG and other neural signals (in banks of up to 64), in the acoustic and higher ranges, that is, with diverse rates starting from 2 KHz, using mainly Innovative Integration (http://www.innovative-dsp.com) DSP-embedded systems (ADC64, M62/7, multiprocessor QUATRO, Chico ). We experimented with recognition of inner tones and have hundreds of recordings with 8-channel EEG, with sampling rates 4–11 KHz. We concentrated mainly on simple experiments. A subject was listening to a calibration tone shortly, then started imagining the same tone, then we had EEG registration for short time, 5–10 seconds. We performed also experiments with simple melodies of external or imagined origin. Our basic tool is Fourier real-time analysis. Examples of the power spectra and spectrograms of EEG recordings of externally played tones, exhibiting the spectral lines corresponding to the played tones, are shown in Figures 1 and 2.

With the inner tones, power spectra and spectrograms are similar to the examples with external tones, but the spectral lines corresponding to the individual tones and their harmonics in the spectrograms are often less prominent or closer to the noise level, hence harder to detect. The complete spectra exhibit a number of features in the HF part of the spectra, not corresponding to the produced inner tones. However, we have positive evidence: in a significant number of experiments (123 out of 147), spectral lines corresponding to imagined inner tones were detected, while the lines corresponding to the tones which were not imagined were not detectable. The experiments with subjects lacking music ability were negative: the tones they imagined were not detectable as the presence of the spectral lines corresponding to the calibration frequencies. We will present some examples with successful extraction of inner tones; more details are available in the mentioned reports. Our signal library and software are available at http://www.matf.bg.ac.yu/~aljosha and http://www.gisss.matf.bg.ac.yu.

## 2. Method

The problem of detection of the inner tones can be seen from two sides. One is when we know which the generated inner tones are, whose traces we are detecting. More difficult is the inverse problem: in the given spectra determine the present inner tones. The complete solution of the former will facilitate solution of the other, which is of importance in the BCI as we propose it. More precisely, we will consider simple tones, that is, those with constant frequency and constant intensity, with a beginning and an end in time. At the beginning all tones could be of the same (similar) length. We call tonal sequence a sequence of simple tones. In this way we omit some of common melodious patterns. A spectrogram of a tonal sequence is a tonal spectrogram. Let us consider a correspondence:

(1) $$f:\;Ts\to S_{\pi },$$

that is, *f* is a correspondence between the space of tonal sequences and the space of tonal spectrograms. For our needs, let *S*_*π*_ be the space of spectrograms of EEG recordings with tonal stimuli of external origin or imagined. We know that *f* cannot be a bijection (hence, the *f* ^−1^ is not a function). However, if we make some restrictions/simplifications on *T*_*s*_, that will have the same effect as introducing an equivalence relation in *T*_*s*_, some sort of glue, identifying certain spectra, which are similar with respect to some properties. Instead of *T*_*s*_, we will be dealing with its homomorphic image. Then, after a reduction of nontonal spectral lines in our EEG spectrograms, we might be able to determine the inverse.

Our initial space *S*_*π*_ consists of the spectrograms of EEG recordings of acoustic stimuli, the tonal sequences, and our basic task is to determine the original tonal sequences from the corresponding spectrograms of EEG recordings. Obviously, the recovery of a tonal sequence is reducible to the sequence of the identifications of individual tones, which simplifies the basic task. Precision constraints are well known in techniques for long time; in the low part, the tonal difference perception, that is, minimum the quarter semitone, determines minimal spectral resolution of 1/4 Hz, while the tonal coloring aliquots have to reach 16 to 20 KHz. Thus, in standard acoustics we need vectors in our simplified spectrograms of up to 80 K coordinates (e.g., the higher quality acoustic standard in broad use is 96 KHz/24bit), adding the number of recording inputs, which is here the number of EEG/MEG electrodes. Hence, we are working in the space whose dimension is beyond 80 000.

For the inner music-based BCI needs, when a subject generates an inner tone, it should be detected and recognized by the BCI. We will introduce simplifications which will reduce this dimension substantially, downscale problem complexity, and bring it closer to be feasible. The composition of all simplifications/restrictions on tonal sequences will define the target homomorphic image of the space *T*_*s*_. But because the nature of music this dimension can hardly go under 4 K. Hopefully, we can neglect a large number of these coordinates at each moment, focusing our attention on the very short subsequences. These are harmonic sequences of individual tones, with <10 aliquots, which have the following form:

(2) $$\langle \lambda_{k}k\nu |\lambda_{k}\varepsilon R,\;\;\;k\varepsilon N\;\cap 10\rangle ,\;\;\text{for}\;\text{a}\;\text{basic}\;\text{frequency}\;\nu ,$$

or with fuzzification:

(3) $$\langle \left[\lambda_{k}k\nu -\delta ,\;\lambda_{k}k\nu +\delta \right]|\lambda_{k}\varepsilon R,k\varepsilon N\cap 10,\delta \varepsilon R\rangle$$

and all have the same length in time. They would form very simple manifolds in those large dimension spaces. Our task is to detect and identify them. Recognition of individual tones of a tonal sequence in the (acoustic) registration of loud singing is simple. The similar task of recognition of an inner (simple) tonal sequence is not so simple and has not been achieved satisfactorily yet.

This approach has some attractive features and leads to some difficulties that may limit its applicability for some time. Generally, we can imagine whatever we can hear. Especially musical contents consisting of consecutive tone series and synchronous tones—intervals and accords. It is simpler to imagine tones to sing mute what can be sung aloud. Our initial restriction to (simple) tonal sequences will be extended by restricting the frequency range to that of a human voice. We have about two and half octaves available as easily controllable (mute) inner tones, that is, the set of about 24 to 32 states. Talented singers control up to 4 octaves, or 48 states, while imaginable tonal interval expands to nearly 100. This gives an opportunity for generation of imagined musical sequences—words, using alphabet of about 30 or more elements. 

Tonal sequences can be produced with similar speed of spoken words. The constraints present in certain tonal sequences roughly correspond to the set of unused sequences in the spoken language. Roughly, with serial tones BCI we are in the range of the verbal communication transfer rates and information flow capacity. Using brain states corresponding to intervals and accords would expand this capacity largely. There are other living species communicating musically and there are natural languages with serious musical components. In either case the development of richer musical languages should follow and would be a nice challenge per se.

## 3. Computational Aspects

Computational aspects will be discussed further with a simple example. Suppose we have two individuals, one producing inner tones in the range c–c2, the other in the range c1–c3 interval. Thus, each is using two octaves. With the tuning fork *a* at 440 Hz, this gives frequency range 132–1056 Hz for both individuals. Suppose that the shortest event time duration corresponds to 1/16th in tempo moderato (ornaments are performed at double and triple speed), which is around 0.2 second. 

The above values set the sampling rate at 2.2 K samples or higher, just to record the first harmonics of the involved tones. Actually the double rate would be necessary. A half quarter tone resolution is needed, which at the lower end of frequency interval gives required spectral resolution of about 2 Hz. The FFT on the input 2 K time series should then provide the desired spectral resolution. The 2 K input FFT covers the time interval of nearly 0.5 second, usually denoted as uncertainty time (because in that time interval the time order is not directly observable from the spectra, which is clear from basic calculations). That means that approximately tone rhythmical values of 1/8th and longer can be located precisely in time. Their amplitudes will be presented correctly. 

In order to resolve shorter rhythmic values and to determine their proper amplitudes, which are essential in the involved inner tones, we would need a recalculation of spectra toward the recalibrated spectra, which can be done easily for the restricted sorts of input tonal sequences, from the obtained spectrograms—time spectra. However, it involves time delay, which is hardly smaller than the time atom. 

**Figure 3:**
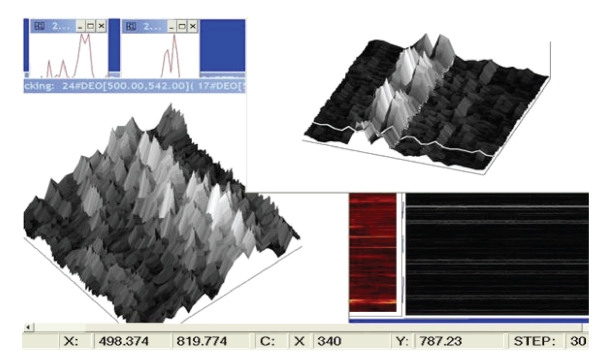
An example of spectrogram feature profiles, magnification of details in the Figure 2, the local neighborhood of c2 and g2 (shown up-787 Hz)—externally played on little organ; both tone profiles show the tonal time stability, but both have spectral width of 15 Hz, while the frequency structure top is stable and reasonably narrow.

Suppose further that we have to deal only with the tonal values from semitonal tempered (classical tonal) system. At the beginning of BCI use, and at any moment after, a calibrating scale can be played. Figure 3 shows how wide in the spectrum could be the externally played tone in an EEG spectrogram. A lot of usual songs satisfy these constrains and simplify further our starting space of tonal sequences *T*_*s*_ for BCI needs. 

Extraction of inner tones may be done in two ways. The first one is to train a neural network to recognize the fingerprints of the inner tones. It can hardly avoid (some sort of) spectrograms as initial objects. This approach is fruitful and can provide easier way to recognize the inner tones. We are experimenting with adaptations of neural networks for speech recognition, developed with the Institute for Applied Mathematics and Electronics (Yugoslav national army/Serbian armed forces), [14]. 

**Figure 4:**
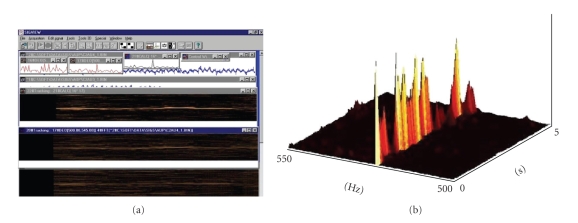
Left side: spectrograms with 800 power spectra parts (frequency interval 500–550 Hz) of two EEG channels with the inner tone c2, lower and middle; their dot product-left top-top view and the side view on the right side-giving the enhanced c2 in the composite spectrogram, well discernible. Time duration 5 seconds.

**Figure 5:**
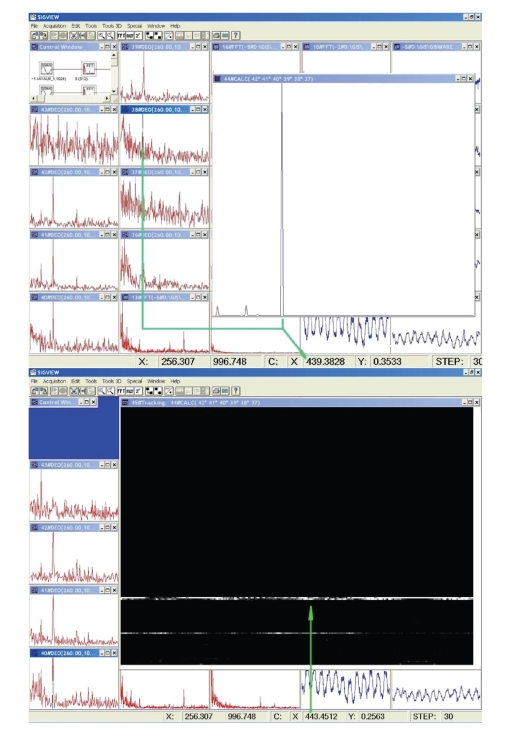
Top: Recording of Inner tone—a tuning fork *a* at 440 Hz, 8 EEG channels, spectral parts from 207 to 2078 Hz, marked 440–443 Hz feature in the dot product of best responding 6 channel spectra with overall well extracted 440–443 Hz line. Lower: (accumulated) time composite spectra—the dot product from the top part of the figure; time horizontal, frequency vertical, intensity coded by brightness.

**Figure 6:**
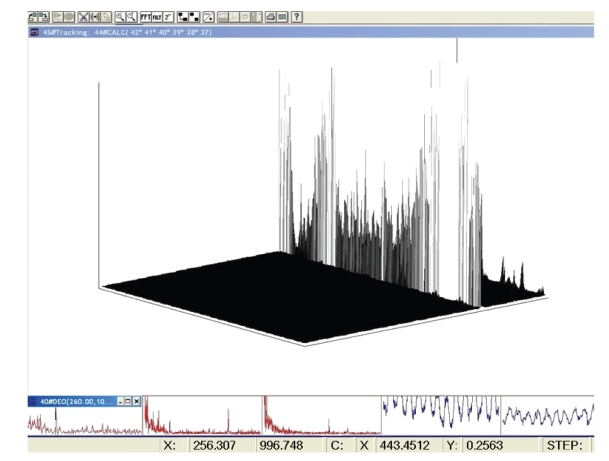
1000 consecutive composite spectra—the dot products of the 6 best responding channels (as in the previous figure top view), the side view, prominent a 440–443 Hz feature-spectral profile, time duration 5 seconds.

Independently, we have developed a system with components of the extractor that include open calculator, with a number of operations on signals and spectra. The inner tone harmonics are present in the signals from different electrodes. Activity recorded with an electrode is partly local. The inner tones harmonics are of smaller magnitude compared to the low frequency (LF) part of the EEG spectra activity, but they are in the HF area. Often, they are hardly discernible in their spectral neighborhood. The spectra are locally linearly dependent in the coordinates with harmonics of inner tones and locally linearly independent in the frequency intervals where the local activity prevails. This means that the composite spectrograms obtained with the dot products of combinations of spectra from different electrodes would enhance the everywhere present spectral lines, which includes the inner tone harmonics, while the spectral zones with prevailing local activity would be zeroed. Some examples with nice spectral localization of inner tones using these properties are presented in Figures 4, 5, and 6.

**Figure 7:**
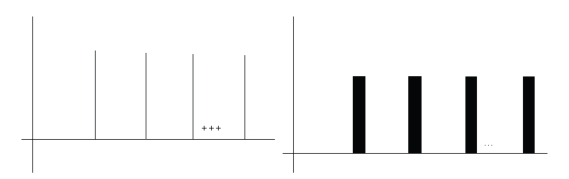
The comb-like tonal representation and its fuzzification are used for the design of the corresponding comb like filters, one for each tone, which support the automatic spectrogram and composite spectrogram analysis using combing operators and subsequent matching measurements within the strategy for the detection of inner tones.

**Figure 8:**
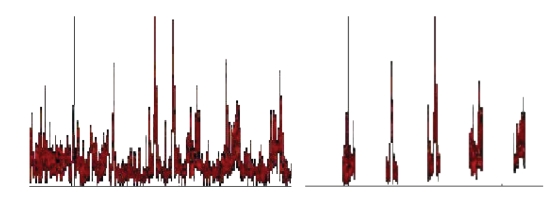
A power spectrum of the externally generated tone c1 on the left; right: the result of its combing with the c1 fuzzy-comb filter in the procedure of measurement of linear dependence between the spectrograms with tonal patterns and the tonal fuzzy comb like filters. The higher the linear dependence, the higher the volume, consequently, the better the matching of spectrogram with a tonal pattern.

**Figure 9:**
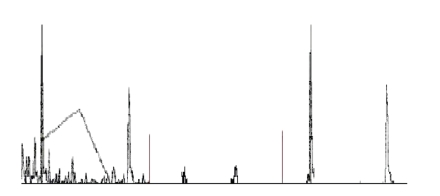
Left: A (part of a) power spectrum of externally sequentially played tones (d, a) (overlapped in the FFT epoch), prominent line corresponding to the tone a; in the middle: the same spectrum combed with the fuzzy comb for c1; right: the same spectrum combed with the fuzzy comb for minor a. This illustrates the response of combing with the wrong and proper matching comb-like filter.

We have implemented comb-like filters and their fuzzifications, corresponding to the tonal structures in (3), at calibrating scale frequencies. These provide a way for an automatic analysis of spectra and composite spectra based on the combing operations and the afterward comparisons with the tonal system-calibrated values, with measurement of best matching, as illustrated with the examples in Figures 7, 8, and 9. This offers a simple strategy and algorithm for the identification of inner tones. The comb-like filters corresponding to the set of tones used for inner singing are coordinate vise multiplied with the spectrograms or composite spectrograms, ordering the outcomes by the maximal volume. We have developed algorithms for the automatic detection of spectrogram feature contours complementing the combing operations.

Next needs are the parallel multiple resolution FFT (which we have in fragments) for short event precise location in time and separation of adjacent tones, feature frequency instability compensation, and separation of tones and their aliquots. 

The tuning system should include a scanning of all channels and a selection of those with better response, a reduction of other HF features not related to external and internal tones, based on the time length discrimination and separation from the calibrating scale tones.

This approach could lead to the intelligent extractor which would be aware of the detected inner tone. In order to improve performance, both approaches can be combined concurrently in parallel. For the further convergence, more experimentation with higher resolution EEG would be necessary. In this way, with proposed steps (some of which are realized), reaching a number of simplifications and partially answering the list of encountered problems, the BCI based on inner tones and inner music would be cured of some deficiencies and instability and will become closer to real applications.

## 4. Discussion

Current BCIs are based on a discrimination of a few commands only. The application of high-resolution EEG in research on inner tones should strongly support further developments of a multicommand system, at least for musically gifted people. It could provide a tool to study causes of musical perception deficiencies, determine and locate problems shared by a large population. It could provide better insight in the difference of musical processing by music professionals and nontrained people, which is highly interesting for cognitive and brain development studies. A number of researchers are successfully involved in inner tones and music [8]. Especially interesting is the recent success of Mick Grierson of Goldsmiths, University of London, who demonstrated high-rate guessing of inner tones with his BCI (reports with real-time show, BBC June 2008, forthcoming [15]). Precise positioning of electrodes will reduce the current complexity of inner tone detection problems with simplified automatic extraction of inner tones and support evolution of the BCI based on inner music [16]. We plan to expand our open system soon with a spatiotemporal analysis and analysis of global trajectories in the transformed space [17]. Other researchers are developing the BCI based on HF EEG [18–20] and further proceeding from biofeedback and with motor imagery-based BCI [20–23].

 The exciting MEG experiments with musical stimuli presented by Andreas Ioannidis in his lecture at the NEUROMATH’2007 workshop in Rome, December 2007, (system and methods presented in [24]), with one millisecond time resolution, demonstrated that a large number of very fast switching interconnected centers are engaged in music processing. This establishes serious hopes that inner music could be subjected to much more sophisticated and sensitive investigation. When we learn more details on mechanisms of this interconnectivity, revealing delays and modulations involved, we might get complementary powerful methods applicable for the study of inner tones, which would result in the improvement of certainty of inner tone detection and representation of details.

## References

[1] Babiloni F, Cincotti F, Marciani M (2007). The estimation of cortical activity for brain-computer interface: applications in a domotic context. *Computational Intelligence and Neuroscience*.

[2] Babiloni F, Cichocki A, Gao S (2007). Brain-computer interfaces: towards practical implementations and potential applications. *Computational Intelligence and Neuroscience*.

[3] Cincotti F, Mattia D, Babiloni C (2002). Classification of EEG mental patterns by using two scalp electrodes and Mahalanobis distance-based classifiers. *Methods of Information in Medicine*.

[4] Cincotti F, Mattia D, Babiloni C (2003). The use of EEG modifications due to motor imagery for brain-computer interfaces. *IEEE Transactions on Neural Systems and Rehabilitation Engineering*.

[5] del R Millan J, Mourino J, Franze M (2002). A local neural classifier for the recognition of EEG patterns associated to mental tasks. *IEEE Transactions on Neural Networks*.

[6] Rosenfeld JP (2000). An EEG biofeedback protocol for affective disorders. *Clinical EEG Electroencephalography*.

[7] Jovanovic A (1998). Brain signals in computer interface. *Intelektualnie Sistemi*.

[8] Zatorre RJ, Halpern AR (2005). Mental concerts: musical imagery and auditory cortex. *Neuron*.

[9] Jovanovic A Inner music, report on the conference Mathematics and other sciences.

[10] Jovanovic A CD-ROM: CCD microscopy, image & signal processing.

[11] Jovanovic A (2001). Research in the group for intelligent systems at Belgrade University, problems and results. *Intelektualnie Sistemi*.

[12] Jovanovic A, Jovanovic M, Perovic A, Maric M A system for neural acoustics analysis.

[13] Jovanovic A, Perovic A (2007). Brain computer interfaces, some technical remarks. *International Journal for Bioelectromagnetism*.

[14] Protic D, Milosavljevic M NNARX model of speech signal generating system: test error subject to modeling mode selection.

[15] Grierson M Composing with brainwaves: minimal trial P300b recognition as an indication of subjective preference for the control of a musical instrument.

[16] Klonowski W (2007). From conformons to human brains: an informal overview of nonlinear dynamics and its applications in biomedicine. *Nonlinear Biomedical Physics*.

[17] Dobosz K, Duch W Fuzzy symbolic dynamics for neurodynamical systems.

[18] Kroger JK, Elliott L, Wong TN, Lakey J, Dang H, George J Detecting mental commands in high-frequency EEG: faster brain-machine interfaces.

[19] Watkins C, Kroger J, Kwong N, Elliott L, George J Exploring high-frequency EEG as a faster medium of brain-machine communication.

[20] Kallenberg M Auditory selective attention as a method for a brain computer interface. http://www.nici.kun.nl/mmm/papers/MK_Auditory_BCI.pdf.

[21] Birbaumer N, Kübler A, Ghanayim N (2000). The thought translation device (TTD) for completely paralyzed patients. *IEEE Transactions on Rehabilitation Engineering*.

[22] Materka A, Byczuk M (2006). Alternate half-field stimulation technique for SSVEP-based brain-computer interfaces. *Electronics Letters*.

[23] Astolfi L, Cincotti F, Babiloni C (2005). Estimation of the cortical connectivity by high-resolution EEG and structural equation modeling: simulations and application to finger tapping data. *IEEE Transactions on Biomedical Engineering*.

[24] Liu L, Arfanakis K, Ioannides A (2007). Visual field influences functional connectivity pattern in a face affect recognition task. *International Journal of Bioelectromagnetism*.

